# U. S. V. M. A.

**Published:** 1895-11

**Authors:** 


### U. S. V. M. A.

The Comitia Minora will decide during the present month the place of meeting for 1896. Among the cities asking for the meeting are Buffalo, N. Y., and Nashville, Tenn. A centennial celebration and exposition will be held in Nashville in September, 1896.

The Comitia Minora for the ensuing year will consist of the regular officers and the following-named members by appointment: Drs. W. L. Williams, Leonard Pearson, Nelson P. Hinkley, L. H. H. Howard, D. E. Salmon, T. B. Rayner, and T. J. Turner.

Dr. S. J. J. Harger has accepted the chairmanship of the Committee on Diseases for the ensuing year.

Dr. J. P. Turner has accepted the post of chairman of the Legislative Committee for the coming year. As one of his associates he will have a very efficient aid in the accepted appointment of Dr. John R. Hart, of Philadelphia.

Dr. Madison Bunker, of Newton, Mass., has accepted the post of Assistant Secretary for that State, and Dr. Warren L. Rhoads a similar place for Pennsylvania.

Veterinarian Osgood's clear, unbiased statements on the subject of tuberculosis won for him the closest attention and warm appreciation of the whole convention.

Another one of the delegates had the misfortune of being detained on his way home by a wreck, fortunately without any injury, though he now counts among his experiences a head-end collision. To add this experience the tunnel this side of Helena proved to be on fire and it became necessary to get out and walk. We wish colleague Williams a better experience next time.

